# Supplementary material for: Vaccination-Route-Dependent Adjuvanticity of Antigen-Carrying Nanoparticles for Enhanced Vaccine Efficacy
Source: Vaccines (Basel). 2024 Jan 26;12(2):125. doi: 10.3390/vaccines12020125 (PMC10892493; doi:10.3390/vaccines12020125)
Supplement: Supplementary file 1 [file vaccines-12-00125-s001.zip › vaccines-2800886-supplementary.pdf]

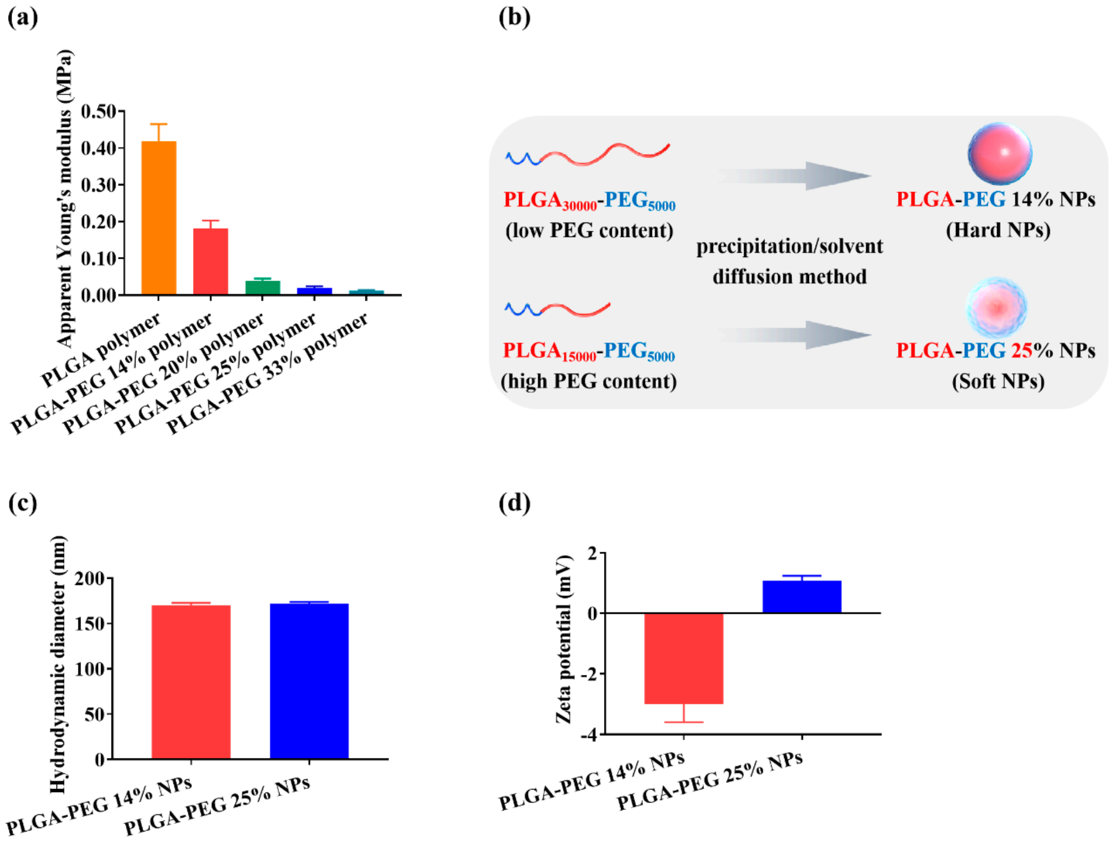

Figure S1. Nano-adjuvant via the precipitation/solvent diffusion method. (a) Apparent Young's modulus of PLGA-PEG X% polymer (X=0, 14, 20, 25, 33) measured by AFM. (b) Schematic illustration of PLGA-PEG X% NPs strategy. (c) Hydrodynamic diameter and (d) Zeta potential of the PLGA-PEG X% NPs measured by DLS (X=14, 25).

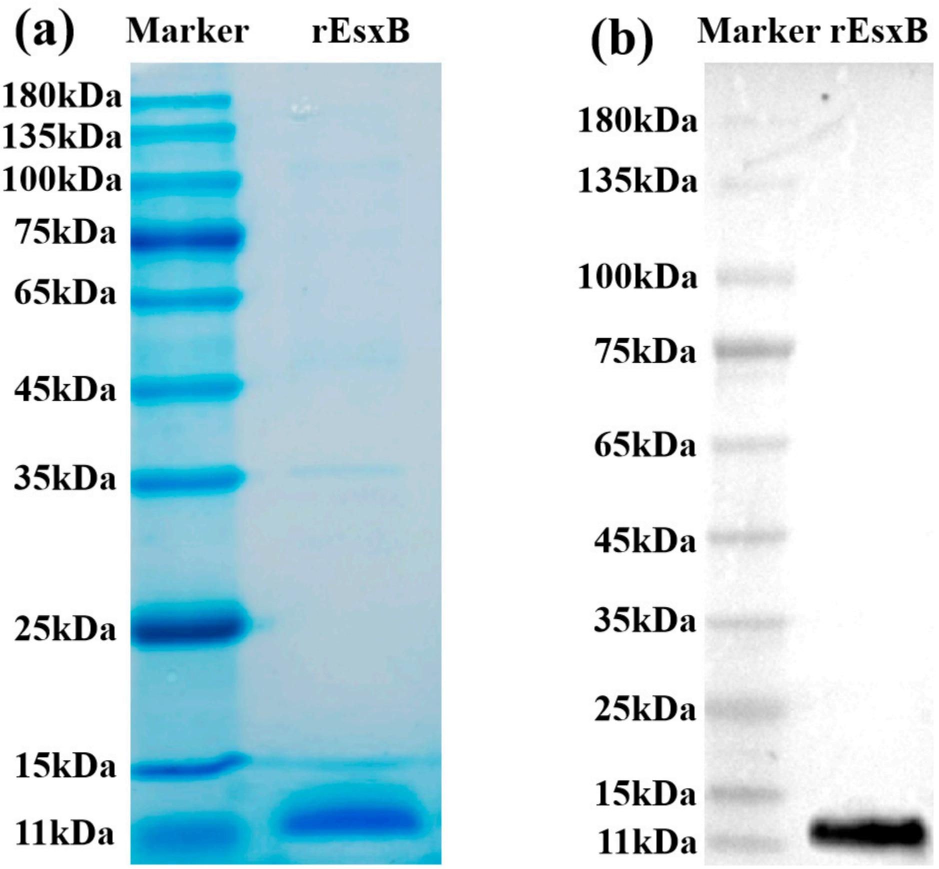

Figure S2. rEsxB antigen was analyzed by (a) SDS-PAGE and (b) Western Blot assay. Lane 1, Marker, Lane 2, 6×His-tag rEsxB. Expected molecular weight of rEsxB is ～11.5 kDa (104 aa).

The purified rEsxB protein was examined, and SDS-PAGE results (**Figure S2a.**) showed a specific band at 11.5kDa, being consistent with the molecular weight of EsxB [34]. The Western Blot analysis (**Figure S2b.**) showed that the purified protein contained 6×His Tag and the predicted molecular weight of rEsxB was the same as that estimated from SDS-PAGE, further confirming its identity.

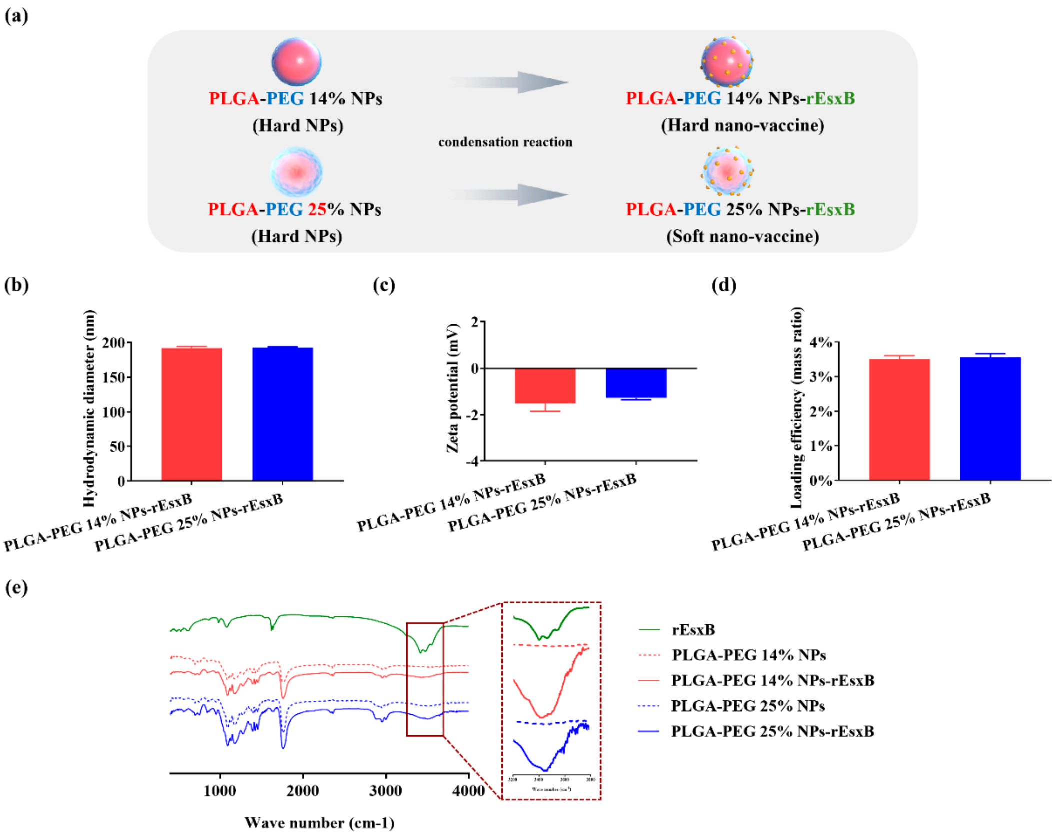

Figure S3. Nano-vaccine via condensation reaction as an enhanced S. aureus vaccine adjuvant. (a) Schematic illustration of nano-vaccine synthesis strategy. (b) Hydrodynamic diameter and (c) Zeta potential of the PLGA-PEG X% NPs-rEsxB measured by DLS. (d) rEsxB loading efficiency of the PLGA-PEG X% NPs-rEsxB measured by BCA. (e) FTIR spectra taken from rEsxB, PLGA-PEG X% NPs, and the PLGA-PEG X% NPs-rEsxB samples (X=14, 25).

In the FTIR spectra taken from samples of rEsxB, NPs of different PEG content, and their corresponding rEsxB conjugates (**Figure S3f.**), the bimodal due to the N-H stretching vibration in $-NH_2$ in the pure rEsxB (~3500 cm^{-1}) disappeared in all of the rEsxB conjugated NPs samples. Instead, the stretching vibration of -NH- (characteristic of the amide bond) can be found, suggesting the formation of amide bond, and thus successful binding of rEsxB to the NPs [35]. On the other hand, all rEsxB conjugated PLGA-PEG X% NPs presented similar surface chemistries, as suggested by their FTIR spectra.

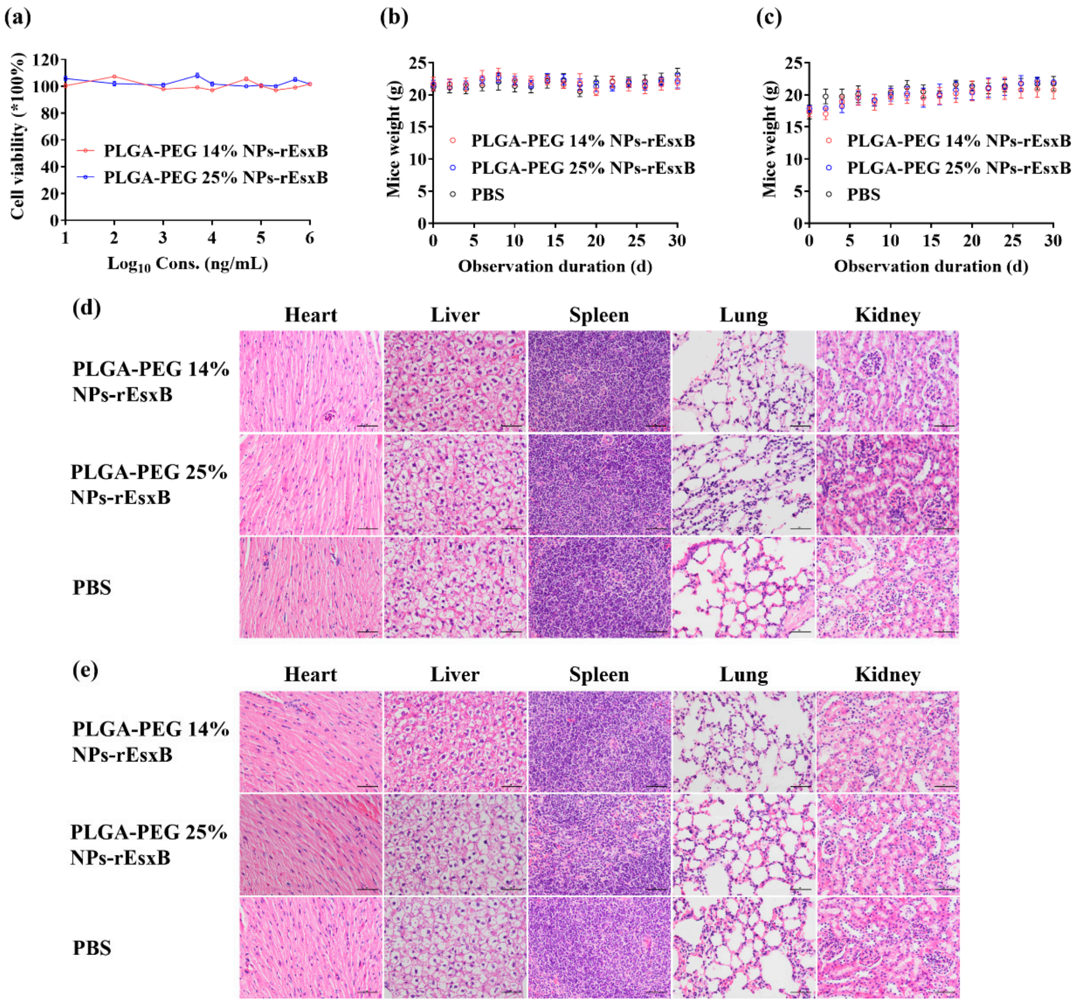

Figure S4. Biocompatibility evaluation of PLGA-PEG X% NPs-rEsxB. (a) Cell viabilities. Body weight change of the mice treated by PLGA-PEG X% NPs-rEsxB, PBS with (b) S.C. and (c) I.V.. H&E staining of major organs of the mice treated by PLGA-PEG X% NPs-rEsxB, PBS with (d) S.C. and (e) I.V. (X=14, 25).

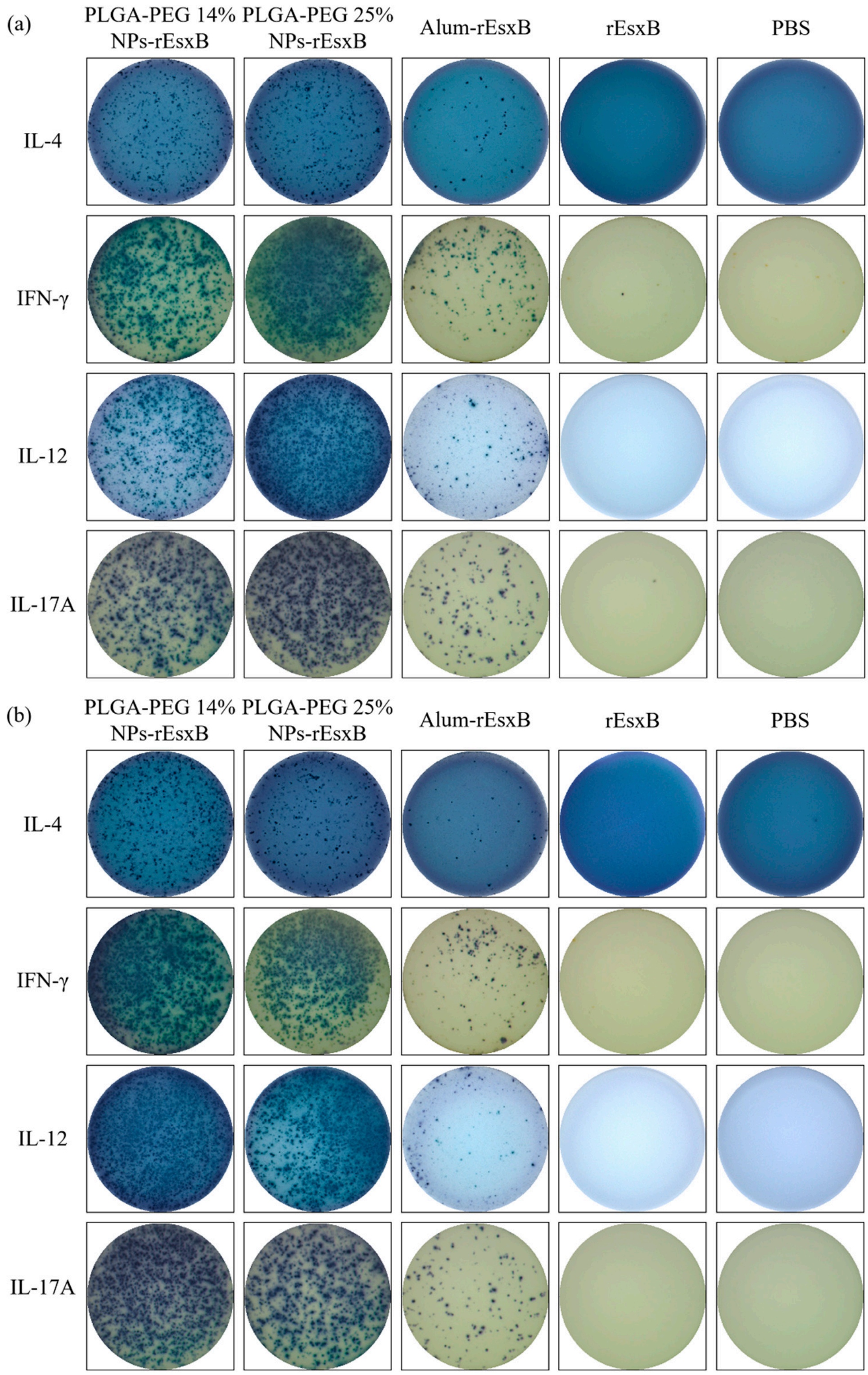

Figure S5. Representative images of ELISPOT wells are shown for cytokines produced by splenocytes with (a) S.C. and (b) I.V..
